# Paracrine Signaling from a Three-Dimensional Model of Bladder Carcinoma and from Normal Bladder Switch the Phenotype of Stromal Fibroblasts

**DOI:** 10.3390/cancers13122972

**Published:** 2021-06-14

**Authors:** Sandra Camargo, Ofer N. Gofrit, Assaf Assis, Eduardo Mitrani

**Affiliations:** 1 Department of Cell and Developmental Biology, The Hebrew University of Jerusalem, Givat Ram, Jerusalem 91904, Israel; sandra.camargo@mail.huji.ac.il (S.C.); assaf.assis@mail.huji.ac.il (A.A.); 2 Department of Urology, Hadassah Medical Center, Faculty of Medicine, The Hebrew University of Jerusalem, Jerusalem 91120, Israel; roferg@hadassah.org.il

**Keywords:** organoids, bladder carcinoma, cancer microenvironment, activated fibroblasts, paracrine signal

## Abstract

**Simple Summary:**

The aim of this work was to create a bladder carcinoma model to study the signals secreted by carcinoma cells in a in vivo-like context and how this molecular signaling can alter stromal fibroblasts. Our model is based on decellularized organ fragments (scaffolds) that aim to preserve the complexity of the native extracellular matrix of the bladder. Primary epithelial cells isolated from human bladder carcinoma were seeded on the scaffolds. We found that those cells showed a similar gene expression pattern when seeded on the scaffolds or on monolayer cultures. However, the secreted pattern of key growth factors was significantly different. Only the combination of factors secreted by carcinoma cells seeded on the scaffolds, but not the carcinoma cells seeded on plastic, was able to induce a pro-inflammatory or myofibroblast phenotype. This model allows one to decipher the paracrine pathways of bladder carcinoma in a defined in vitro system.

**Abstract:**

We present a three-dimensional model based on acellular scaffolds to recreate bladder carcinoma in vitro that closely describes the in vivo behavior of carcinoma cells. The integrity of the basement membrane and protein composition of the bladder scaffolds were examined by Laminin immunostaining and LC–MS/MS. Human primary bladder carcinoma cells were then grown on standard monolayer cultures and also seeded on the bladder scaffolds. Apparently, carcinoma cells adhered to the scaffold basement membrane and created a contiguous one-layer epithelium (engineered micro-carcinomas (EMCs)). Surprisingly, the gene expression pattern displayed by EMCs was similar to the profile expressed by the carcinoma cells cultured on plastic. However, the pattern of secreted growth factors was significantly different, as VEGF, FGF, and PIGF were secreted at higher levels by EMCs. We found that only the combination of factors secreted by EMCs, but not the carcinoma cells grown on plastic dishes, was able to induce either the pro-inflammatory phenotype or the myofibroblast phenotype depending on the concentration of the secreted factors. We found that the pro-inflammatory phenotype could be reversed. We propose a unique platform that allows one to decipher the paracrine signaling of bladder carcinoma and how this molecular signaling can switch the phenotypes of fibroblasts.

## 1. Introduction

We developed a three-dimensional in vitro model to recreate bladder carcinoma in vitro that aims to recapitulate the in vivo behavior of carcinoma cells and how they are found in their natural tissue environment. 

During development, the interaction between parenchymal cells and their stroma is fundamental in determining the final fate of the epithelial cells [1]. In adult tissues, a well-established two-way communication between cells and their surroundings leads to the correct function and dynamics of the organ. The protein composition, cellular composition, and architecture of the stroma are essential to maintain tissue homeostasis [1]. When this equilibrium is disrupted, it can produce important structural, physical, and biochemical changes that promote carcinogenesis [1,2,3]. Clearly, there is a positive and/or negative reciprocal regulation between malignant epithelial cells and the local tumor microenvironment (TME) [1,3]. The development of ever-more realistic in vitro models to study the epithelium–stroma communication and the role of the microenvironment during carcinogenesis has received further recognition due to its relevance for drug-screening in personalized medicine and, of course, because it is fundamental for understanding the basic biology of cancer.

Tumors are complex and dynamic tissues where both cellular and noncellular components from the tumoral niche have important roles. The protein composition and structure of the extracellular matrix (ECM) not only maintain homeostasis in normal tissues but also play key roles during carcinogenesis [4]. To model cancer in vitro, several kinds of synthetic biomaterial matrices, such as polymers; natural matrices, such as porcine intestinal submucosa, collagen, or cell-derived extracts; and other proteins extracted from the extracellular matrix have been used as scaffolds for 3D cultures [5]. Recent 3D cancer models have been based on organoids and spheroids. They have been widely used and have demonstrated cell proliferation, organization, and differentiation with the addition of growth factors [5,6,7,8]. The 3D structure provided by these types of cultures allows for better cell function and differentiation than when the cells are seeded in 2D in standard plastic culture dishes [9].

In addition to the ECM, stromal cells such as fibroblasts, endothelial cells, and cells of the immune system are key players in restricting or promoting tumor progression [5,10,11]. Specifically, activated fibroblasts present in the TME, known as cancer-associated fibroblasts (CAFs), have key roles in matrix remodeling, angiogenesis, and the immune response during carcinogenesis, metastasis, and drug resistance [12]. For 3D in vitro cancer models, there has been an attempt to recreate the interaction of tumoral epithelial cells with both the extracellular matrix and the stromal cells to elucidate the signaling led by each component. Many studies have described the different subpopulations of activated fibroblasts present in the TME while trying to decipher their role, heterogeneity, and plasticity [12]. It has been demonstrated that fibroblasts can switch between different functional states, and three phenotypes have been reported: a matrix-producing contractile phenotype called the myofibroblast-CAF, an immunomodulator phenotype known as the inflammatory CAF, and (recently) a subpopulation that expresses MHC class II and CD74+ called the antigen-presenting CAF [12,13,14,15].

We took advantage of a 3D in vitro system developed by our group based on natural decellularized organs of microscopic dimensions that conserve the micro-architecture and protein composition of the original organ [16,17,18] to recreate an in vitro carcinoma [19]. The purpose of this system is to recreate, much as possible, as the in vivo conditions where epithelial–stromal interactions are preserved in an in vitro system. Our model falls into the organoid category, though with a higher level of complexity where cells are seeded on scaffolds that closely preserve the composition and structure of the native organ.

We have also used another 3D platform developed in our laboratory based on small organ fragments whose geometry allows for the preservation of the natural epithelial/mesenchymal interactions [20]. This technology allows us to maintain fragments of normal human bladder in vitro.

These two systems allowed us to further analyze the factors secreted by normal and carcinoma epithelial cells from the human bladder in an in vivo-like environment and study their effects on modulating fibroblast phenotypes.

## 2. Materials and Methods

### 2.1. Preparation of Decellularized Bladder Scaffolds

Bladder specimens were obtained from pig bladders. The decellularization process was done following a previously described protocol [16,17,18,19], with the following modifications. The smooth muscle layer was removed by surgical delamination and was then cut into 1 cm^2^ fragments. After cutting, the fragments were processed under continuous shaking in 200 mL of the following solutions: four washes with 1 M NaCl for 20 min each, four short 3 min washes with distilled water, and three washes with 0.5% Triton-X100 for 20 min each. Then there were six washes with distilled water for 20 min each and one wash with PBS supplemented with 1% penicillin (100 U/mL)–streptomycin (1 mg/mL) and 1 mL of gentamicin (50 mg/mL) for 20 min. Bladder scaffolds were stored in PBS supplemented with 1% penicillin (100 U/mL)–streptomycin (1 mg/mL) and 1 mL of gentamicin (50 mg/mL) overnight at 37 °C to allow for RNA fragment degradation, and they were finally stored at 4 °C.

### 2.2. Nano LC–MS/MS Analysis

MS/MS analysis was performed using a Q Exactive-HF mass spectrometer (Thermo Fisher Scientific, Waltham, MA, USA) coupled on-line to a Nanoflow UHPLC instrument, Ultimate 3000 Dionex (Thermo Fisher Scientific). Peptides dissolved in 0.1% formic acid were separated without a trap column over a 120 min acetonitrile gradient run at a flow rate of 0.3 μL/min on a reverse phase 25-cm-long C18 column (75 μM ID, 2 μM, 100 Å, Thermo PepMap RSLC, Waltham, MA, USA). Survey scans (300–1650 *m*/*z*; target value: 3 × 10^6^ charges; maximum ion injection time: 20 milliseconds) were acquired and followed by higher energy collisional dissociation (HCD)-based fragmentation (normalized collision energy 27). A resolution of 60,000 was used for survey scans, and up to 15 dynamically chosen most abundant precursor ions with “peptide preferable” profiles were fragmented (isolation window 1.6 *m*/*z*). The MS/MS scans were acquired at a resolution of 15,000 (target value 1 × 10^5^ charges, maximum ion injection times 25 milliseconds). Dynamic exclusion was 20 s. Data were acquired using Xcalibur software (Thermo Scientific). To avoid a carryover, the column was washed with 80% acetonitrile and 0.1% formic acid for 25 min between samples (done at the Interdepartmental Instrumentation Facility at the Hebrew University of Jerusalem, Jerusalem, Israel).

### 2.3. MS/MS Data Analysis

Mass spectra data were processed using the MaxQuant computational platform, version 1.6.14.0. Peak lists were searched against the relevant UniProt FASTA sequence databases: porcine (Sus scrofa) from 17 May 2020 containing 120,834 entries. The search included cysteine carbamidomethylation as a fixed modification and the oxidation of methionine as variable modifications, and it allowed for up to two miscleavages. The match-between-runs option was used. Peptides with a length of at least seven amino acids were considered, and the required FDR was set to 1% at the peptide and protein levels. Protein identification required at least 2 unique or razor peptides per protein. Relative protein quantification in MaxQuant was performed using the label-free quantification (LFQ) algorithm [21].

### 2.4. Cell Culture

Primary urothelial cells were isolated from normal human bladder (1 donor) and human bladder carcinoma (3 donors; see Table S1 for more information) under the approval of the Ethical committee for Medical Research of Hadassah Hospital (IRB # 0279-18-HMO).

Bladder samples were collected in Dulbecco’s Modified Eagle’s Medium (DMEM). Then, samples were washed three times with DMEM supplemented only with 1% penicillin (100 U/mL)–streptomycin (1 mg/mL). After washes, samples were cut with a scalpel into small fragments between 0.2 and 0.5 mm^2^ and put into 1 mL of 0.25% trypsin (four tissue fragments per tube) at 37 °C for 30 min. All supernatants were mixed and collected to be neutralized with DMEM, and they were supplemented with 10% fetal calf serum, 1% penicillin (100 U/mL)–streptomycin (1 mg/mL), and 1% L-glutamine (2 mM) (supplemented DMEM). Then, they were centrifuged at 1800 rpm for 5 min. Recovered cells were directly seeded onto decellularized bladder scaffolds as described below. Simultaneously, some fragments were placed in standard culture dishes and adhered to the surface to allow cells to emerge from the tissue. These cells were used as control carcinoma cells grown on plastic. For cells grown on bladder scaffolds or standard culture dishes, supplemented DMEM was used. All reagents were from Biological Industries, Israel. The medium was changed every 2–3 days. No test for *Mycoplasma* was done.

Primary bladder fibroblast from normal tissue (ATCC PCS-420-013) were maintained in supplemented DMEM. The medium was changed every 2–3 days. Passages 2–7 were used for the experiments. No test for *Mycoplasma* was done.

### 2.5. Preparation of Bladder Engineered Micro-Carcinomas (EMCs)

Stored scaffolds were first washed three times in PBS, and each was inserted into a well on a 24-well plate (1 scaffold/well). The stromal side of each scaffold was placed in touch with the well surface without medium. Primary epithelial cells isolated from human bladder carcinoma were seeded on each scaffold by carefully layering 100 µL of culture media containing cells (1 × 10^5^ cells/scaffold) on the basal side of the scaffold (engineered micro-carcinomas (EMCs)). The EMCs were incubated at 37 °C and 5% CO_2_ for 3 h. Supplemented DMEM was added at 400 µL/well and incubated at 37 °C in 5% CO_2_. The medium was changed every 2–3 days. The media from the cultures were collected (conditioned medium) and kept at −20 °C for further analysis.

### 2.6. Normal Human Bladder Micro-Organs

A method for preparing micro-organs was previously described [20]. Briefly, a normal human bladder was taken from the tissue immediately surrounding a tumor from one donor and maintained in PBS. Then, the tissue was cut transversely into 300-μM-thick per 1 cm^2^ length fragments using a Sorvall TC-2 tissue chopper and then again put in PBS. After cutting, the resulting normal bladder micro-organs were washed three times with PBS and 2% penicillin (100 U/mL)–streptomycin (1 mg/mL). Then, micro-organs were transferred to a 48-well-plate (5 micro-organs per well in 250 μL) and maintained in supplemented DMEM without fetal calf serum at 37 °C in 5% CO_2._ The medium was changed every 2–3 days. The media from the cultures were collected (conditioned medium) and kept at −20 °C for further analysis.

### 2.7. Conditioned Media Analysis

The conditioned media from EMCs, cells on plastic, and normal bladder micro-organs were collected, centrifuged at 1800 rmp, and filtered through a 0.22 μM filter to remove any cell debris. The detection of growth factors in each conditioned medium was done using the quantitative Human Angiogenesis Antibody Array (Abcam, Cambridge, UK). For the analysis of protein concentration per number of cells, each concentration of obtained protein was normalized to 1 × 10^5^ cells using the number of cells obtained based on the extracted RNA and under the assumption that 1 µg of RNA represents 1 × 10^5^ cells.

### 2.8. Activation of Fibroblasts

Primary bladder fibroblast from normal tissue (ATCC PCS-420-013) were seeded in supplemented DMEM at a concentration of 3 × 10^3^ cells/well in a 96-well plate and maintained at 37 °C in 5% CO_2_. After 24 h of incubation, cells (80–100% of confluence) were washed with PBS and exposed to different concentrations of the conditioned medium (100%, 50%, and 25%; dilutions were done using supplemented DMEM), of EMCs, and of cells on plastic and from normal bladder tissue (CM-EMC, CM-cells on plastic, CM-normal tissue, respectively), and then they were incubated for another 24 h at 37 °C in 5% CO_2_. Fibroblasts only exposed to supplemented DMEM were used as control. After incubation, cells were washed with PBS and collected for RNA extraction following the protocol of Tri-Reagent (Sigma, Rehovot, Israel). Afterwards, qRT-PCR was done using the Fast SYBR^TM^ Green Master Mix Kit protocol (Applied Biosystems, Foster City, CA, USA), as described below.

### 2.9. Reversal of the Activated Phenotype

As described above, primary bladder fibroblast from normal tissue (ATCC PCS-420-013), were seeded first in supplemented DMEM at a concentration of 3 × 10^3^ cells/well in a 96-well plate and maintained at 37 °C in 5% CO_2_; after 24 h of incubation, fibroblasts were treated with 100% CM-EMC and 100% CM-normal tissue. After another 24 h of incubation, fibroblasts first treated with 100% CM-EMC were re-treated with 100% CM-normal tissue and fibroblasts first treated with 100% CM-normal tissue were re-treated with 100% CM-EMC. In addition, the other three groups of fibroblasts were treated as follows: 100% CM-EMC, 100% CM-normal tissue, or DMEM, supplemented with 10% FCS. After 24 h of incubation with the last treatment, cells were washed with PBS and collected for RNA extraction following the protocol of Tri-Reagent (Sigma, Rehovot, Israel). Afterwards, qRT-PCR was done using the Fast SYBR^TM^ Green Master Mix Kit protocol (Applied Biosystems, Foster City, CA, USA), as described below (Section 2.13).

### 2.10. DAPI Staining

Bladder fragments, decellularized matrices (Figure S1A,B), and EMCs were fixed in 4% paraformaldehyde, and then nuclei were stained with 5 μg/mL of 4-6-diamino-2-phenylindole dihydrochloride (DAPI) (Sigma, Rehovot, Israel) in PBS.

### 2.11. H&E Staining

EMCs were fixed 4% paraformaldehyde and embedded in paraffin; then, 8 μM sections prepared and stained with hematoxylin/eosin (H&E) according to routine histological methods.

### 2.12. Immunostaining

Bladder scaffolds (300-µM-thick fragments) were fixed 4% paraformaldehyde and then kept at 4 °C in 1% formaldehyde in sealed containers in preparation for processing. Immunostaining was done using the anti-Laminin, primary rabbit antibody (Sigma, Rehovot, Israel) diluted 1:200 in 2% BSA in PBS overnight at 4 °C. The secondary, donkey anti-rabbit antibody (Sigma, Rehovot, Israel) was used on the second day of staining.

### 2.13. Relative Quantitative Real-Time Polymerase Chain Reaction

The extraction of RNA from tissue fragments, EMCs, or cells seeded on plastic was done following the protocol of Tri-Reagent (Sigma, Rehovot, Israel). Then, cDNA preparation was done according to the Reverse Transcription Kit (Applied Biosystems, Foster City, CA, USA). Afterwards, qRT-PCR was done using the Fast SYBR^TM^ Green Master Mix Kit protocol (Applied Biosystems, Foster City, CA, USA) as follows: 10 μL of 2X SYBR^®^ Green PCR Master Mix, 2 μL of primer mix (10 μM of forward primer and 10 μM of reverse primer), 6 μL of ultrapure water (Sigma, Rehovot, Israel), and 2 μL of cDNA were used to reach a final volume of 20 μL per well in a 96-well multiplate.

Expression levels were calculated by the ΔCT method after normalizing the genes with TATA-box binding protein (TBP) as follows: for experimental samples (cells seeded on scaffolds—EMCs) and the experimental control (cells seeded on plastic dishes), the average of each target gene CT was normalized with the average of the housekeeping gene CT for each time point. At this point, there are two values: the delta CT value of the target gene for experimental samples (ΔCTS) and the delta CT value of the target gene for the experimental control (ΔCTC). Then, the difference between ΔCTS and ΔCTC was calculated as the double delta CT value (ΔΔCT). Finally, the value of 2^−ΔΔCT^ was calculated to get the expression fold change. Pre-designed primers specific to human sequences for TBP, AKT, BCLXL, MYC, MET, PTEN, FAS, IL6, IL1, FAP, αSMA, vascular endothelial growth factor (VEGF), and fibroblast growth factor (FGF) were purchased from Sigma (KiCqStart SYBR Green Primers).

### 2.14. Statistical Analyses

Statistical analysis was performed using *t*-test and based on the data of two biological replicates and at least two technical replicates. A *p*-value of ≤0.05 was considered significant, and a *p*-value of ≤ 0.01 was considered highly significant.

## 3. Results

### 3.1. Characterization of Bladder-Derived Scaffolds

**Figure 1:**
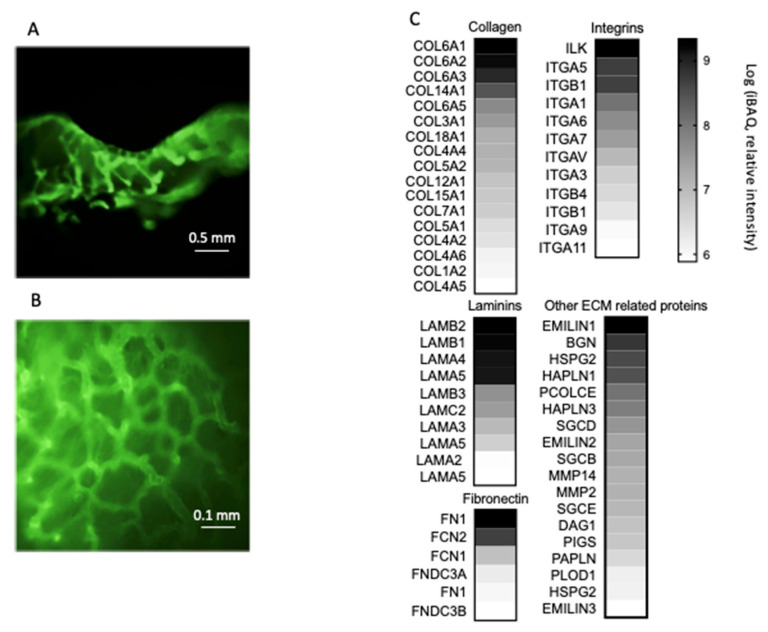
Characterization of the extracellular matrix from bladder-decellularized matrices. The basement membrane was intact, as manifested by laminin staining: (**A**) side view and (**B**) top view. (**C**) Selected proteins from the extracellular matrix of the bladder scaffolds. iBAQ: intensity-based absolute quantification.

We obtained bladder-derived scaffolds from pigs with the aim of preserving the structure of the original ECM of the bladder, as well as ECM-associated growth factors. To that extent, the tissues were cut into 1 cm^2^ pieces and decellularized as previously described. After the decellularization of bladder fragments, the integrity of the basement membrane was examined by immunostaining with Laminin antibodies (Figure 1A,B). The protein composition was analyzed by LC–MS/MS. Figure 1C shows that the matrix was rich in several types and quantities of collagen, integrin, laminin and fibronectin, and it also contained characteristic bladder proteins such as the elastin microfibril proteins EMILIN1, EMILIN2, and EMILIN3. Additional information regarding other abundant proteins present in the decellularized matrices is shown in Figure S1C.

### 3.2. Bladder Engineered Micro-Carcinomas (EMCs) Using Human Primary Bladder Carcinoma Epithelial Cells

**Figure 2:**
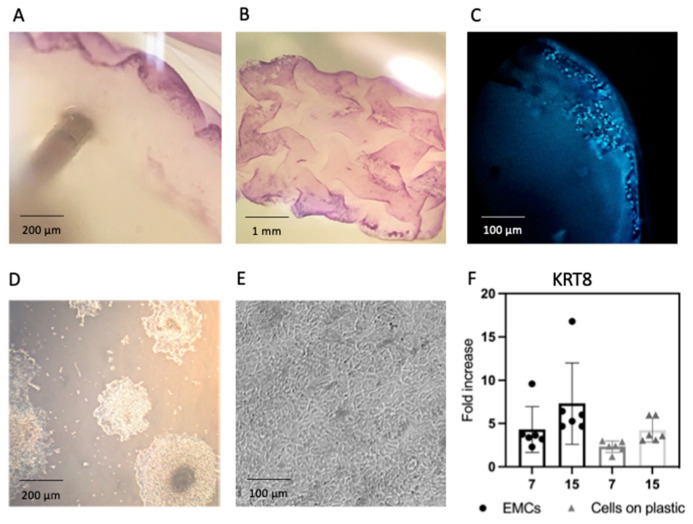
Primary epithelial cells derived from human bladder carcinoma were found to adhere to the scaffold basement membrane. Human primary urothelial cells from bladder carcinoma cultured on bladder scaffolds stained with MTT after (**A**) 7 days and (**B**) 15 days, and they were stained with (**C**) DAPI after 7 days. Human primary urothelial cells from bladder carcinoma cultured on plastic after (**D**) 7 days and (**E**) 15 days. (**F**) Cells grown on plastic or bladder scaffolds expressed the tissue specific marker KRT8. Fold increase of samples was calculated by normalizing with TBP and original bladder carcinoma at day 0 was used as control. Scale bars represent 200 µm for (**A**) and (**D**), 1 mm for (**B**), and 100 µm (**C**,**F**).

In order to create a more realistic system, the bladder engineered micro-carcinoma model was developed using primary epithelial cells isolated from human bladder carcinoma and seeded onto bladder scaffolds (EMCs). Primary urothelial cells were isolated from human bladder carcinoma biopsies from three different donors. Figure 2A–C and Figure S2B suggest that primary carcinoma cells adhered to the scaffold basement membrane and created a contiguous one-layer epithelium. In order to obtain primary cultures on plastic dishes to be used as controls, the biopsies were cut in small fragments and adhered to the surface of standard cultures dishes, as described in the methods section. Figure 2D shows cells emerging from fragments and adhering to the culture dishes on day 7. Figure 2E shows a resulting monolayer on day 15 (the maximum time tested). Figure S2A shows the number of cells per scaffold on days 7 and 15. Both the cells that eventually grew on plastic and those that seeded on bladder scaffolds expressed the tissue-specific marker KRT8, as shown in Figure 2F.

**Figure 3:**
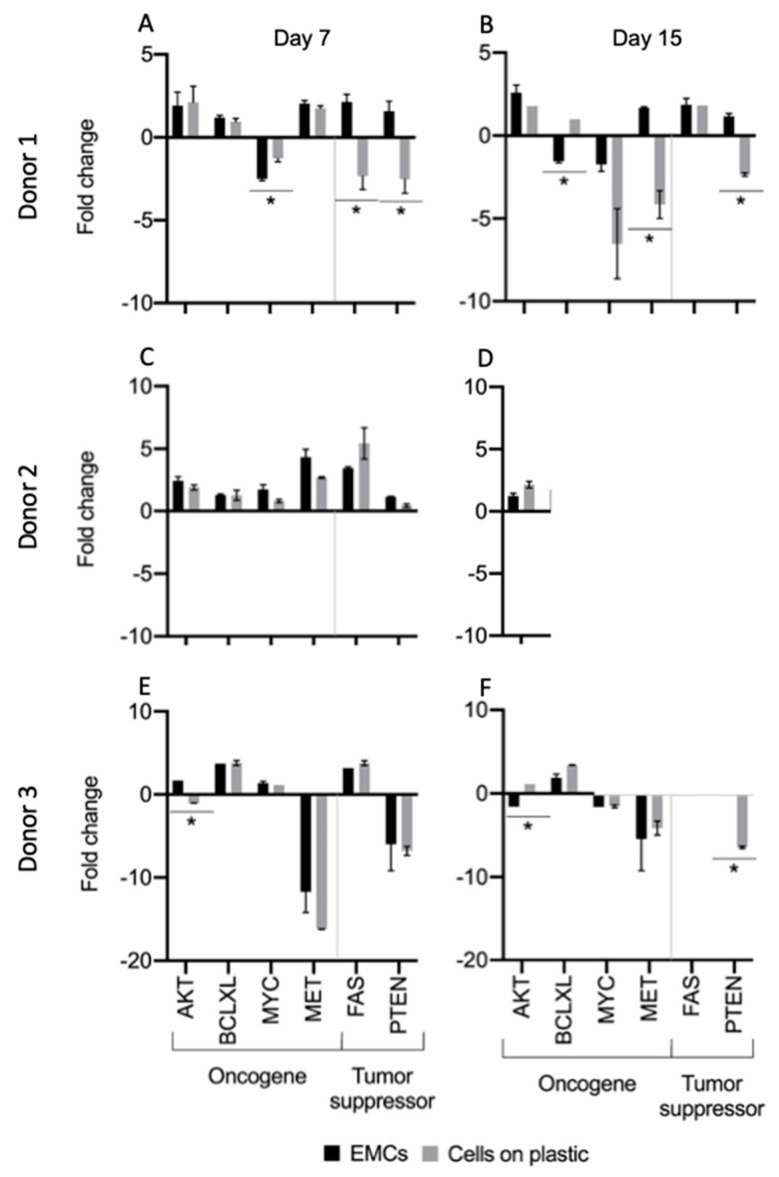
The gene expression pattern of primary carcinoma cells derived from human bladder carcinoma seeded on bladder scaffolds (EMCs) was similar to the profile of the original carcinoma on day 0 and to the pattern of the same cells seeded on plastic. Primary epithelial cells from bladder carcinoma isolated from: (**A**,**B**) donor 1, (**C**,**D**) donor 2, and (**E**,**F**), donor 3 on days 7 and 15. The fold change of samples was compared with the original bladder carcinoma on day 0. * Statistically significant *p* ≤ 0.05.

Contrary to what happens when primary normal cells are grown in the scaffold as compared to the cells grown in plastic, where the gene expression profile is significantly different 16,17, the gene expression of the genes tested displayed by EMCs was remarkably similar to the pattern expressed by the (naked) cells cultured on plastic at day 7 and 15 (Figure 3). The expression of the tested EMCs genes was more similar to the gene expression pattern of the original tumor than to the gene expression pattern of primary carcinoma cells seeded on plastic.

**Figure 4:**
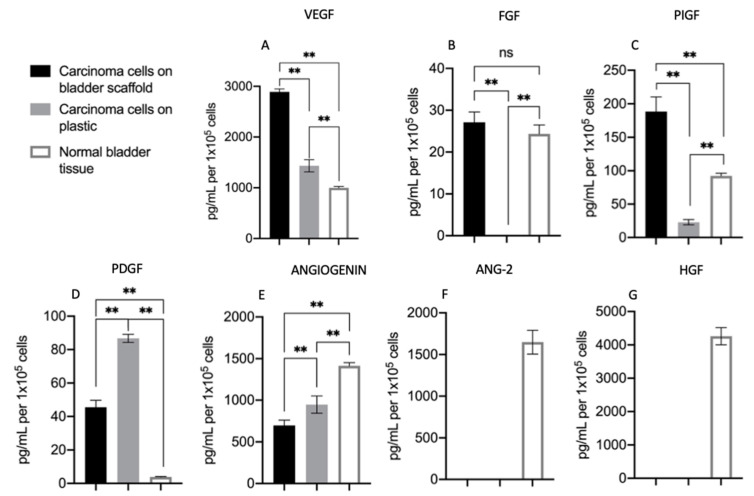
Different patterns of secreted growth factors of primary bladder carcinoma cells seeded on bladder scaffolds or on plastic, as well as of normal bladder tissue: (**A**) vascular endothelial growth factor (VEGF), (**B**) fibroblast growth factor (FGF), (**C**) placental growth factor (PIGF), (**D**) platelet-derived growth factor, (**E**) angiopoietin 2 (ANG-2), (**F**) angiogenin, and (**G**) hepatocyte growth factor (HGF). ** Statistically highly significant *p* ≤ 0.01; ns: no significant.

However, the pattern of secretion of some key growth factors was different. VEGF, FGF, and placental growth factor (PIGF) were significantly higher in EMCs than in cells seeded on plastic (Figure 4). All of the factors found to be upregulated are known for being involved in tumor neovascularization and vascular remodeling [22,23]. In addition, PIGF, which was also found to be highly secreted by EMCs, is known to be involved in the tumoral immune response [22,23].

For the normal human bladder cultures, it should be noted that the pattern of secreted growth factors was significantly different from the secretion patterns found on both the EMCs cultures and primary carcinoma cells grown on plastic (Figure 4). Specifically, two additional growth factors, angiopoietin 2 (ANG-2) and hepatocyte growth factor (HGF) (Figure 4F,G, respectively), as well as VEGF and PIGF, were secreted at lower levels by normal bladder cultures than by EMCs (Figure 4A,C, respectively).

Even when the gene expression of the tested genes was very similar in the carcinoma cells seeded on the bladder scaffolds and on plastic, it was possible to establish a different phenotype between them with the pattern of growth factors secreted into the conditioned medium. Clearly, the behavior of carcinoma cells when grown in their natural microenvironment (EMCs) is different than when carcinoma cells are grown on plastic and probably more accurately reflects what is happening in vivo.

### 3.3. Factors Secreted from Bladder Engineered Micro-Carcinomas But Not the Naked Carcinoma Cells Activate Normal Stromal Fibroblasts

**Figure 5:**
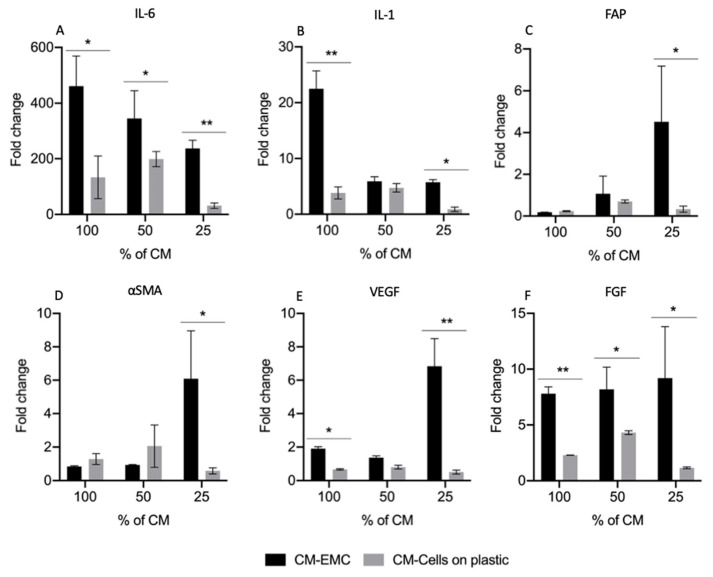
Different concentrations of conditioned medium from bladder-EMC cultures could induce two different phenotypes on fibroblasts. First was a pro-inflammatory phenotype with the upregulation of (**A**) IL-6 and (**B**) IL-1. Second was myofibroblast phenotype with the upregulation of (**C**) FAP, (**D**) αSMA, (**E**) VEGF, and (**F**) FGF. * Statistically significant *p* ≤ 0.05. ** Statistically highly significant *p* ≤ 0.01.

Normal primary fibroblasts derived from human bladder were first exposed to the collected conditioned medium on days 6, 10, 15, and 30 from EMCs cultures and cultures of the same primary carcinoma cells seeded on plastic (Figure S3). Because a more pronounced effect on fibroblasts was observed when they were exposed to the CM from cultures on day 10, fibroblasts were exposed to different concentrations of CM on day 10 (100%, 50%, and 25%). As shown in Figure 5, the gene expression profile of fibroblasts was significantly different when treated with CM from EMCs (CM-EMC) and CM from cells on plastic compared with the fibroblast control grown in standard DMEM with 10% FCS. The CM-EMC at a concentration of 100% induced a significant upregulation of pro-inflammatory cytokines (interleukin 6 (IL-6) and interleukin 1 (IL-1)) on normal-bladder stromal fibroblasts, thus suggesting a pro-inflammatory phenotype (Figure 5A,B). This phenotype was not expressed on fibroblasts exposed to CM from naked carcinoma cells seeded on plastic. Interestingly, when the CM-EMC concentration was lowered to 25%, the gene expression profile of fibroblasts showed a different pattern of fibroblast markers. In this case, fibroblast activation protein (FAP), alpha smooth muscle actin (α-SMA), VEGF, and FGF were upregulated, as shown in Figure 5C–F. This fibroblast gene expression profile induced with 25% of CM-EMC suggested a shift to a myofibroblast phenotype that was different to the phenotype displayed by the fibroblasts when exposed to the highest concentration of CM-EMC. Overall, these changes in the gene expression at different concentrations of CM were only observed in fibroblasts exposed to CM-EMC and not in the same fibroblasts exposed to CM from carcinoma cells seeded on plastic.

### 3.4. Factors Secreted from Cultures of Normal Bladder Reverse the Pro-Inflammatory Phenotype of Activated Fibroblasts

Once it was established that 100% CM-EMC activated normal stromal fibroblasts into a pro-inflammatory phenotype and under the hypothesis that “benign signaling” from normal tissue would not activate fibroblasts or could reverse activated fibroblast, new tests where fibroblasts were exposed to CM from cultures of normal bladder were performed.

Normal stromal fibroblasts from human bladder were exposed to four different conditions. The first and second groups of fibroblasts were only exposed to one treatment with 100% CM-EMC and 100% CM from normal bladder cultures, respectively. The third group was exposed to 100% CM-EMC for 24 h and then to 100% CM from normal bladder cultures for another 24 h. The last group was first exposed to 100% CM from normal bladder cultures for 24 h and then to 100% CM-EMC for another 24 h.

**Figure 6:**
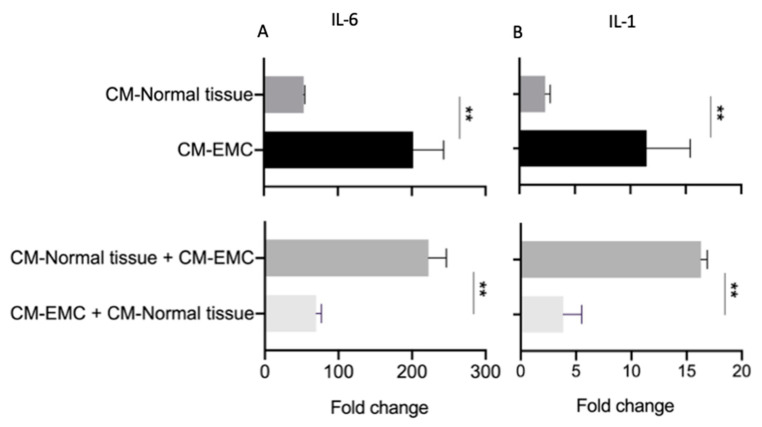
A paracrine signal from cultures of normal bladder tissue could reverse the pro-inflammatory phenotype induced on fibroblasts exposed to the paracrine signaling from bladder-EMC cultures. (**A**) Gene expression of IL-6 and (**B**) IL-1 on fibroblasts exposed to the conditioned medium of bladder-EMC cultures or normal bladder cultures compared with fibroblasts cultured in DMEM with 10% FCS. ** Statistically highly significant *p* ≤ 0.01.

The first group confirmed that normal fibroblasts could be activated by the secreted signaling from EMCs. The second group showed that even when the secreted signaling from normal tissue could induce an upregulation of IL-6 and IL-1 compared with the control of fibroblasts grown in standard DMEM with 10% FCS, the upregulation of those cytokines was significantly lower than in the group treated with 100% CM-EMC (Figure 6). Together, these results suggest that fibroblasts exposed to each condition displayed a different phenotype.

Finally, in the third group, we showed that after activating fibroblasts with 100% CM-EMC and then exposing them to a “normal signaling,” these fibroblasts show a phenotype similar to the group treated only with 100% CM from normal bladder cultures (Figure 6). In addition, the fibroblasts that were first exposed to 100% CM from normal bladder cultures and then to 100% CM-EMC displayed a gene expression pattern similar to the fibroblasts treated only with 100% CM-EMC (Figure 6). A control group of activated fibroblasts with 100% CM-EMC was exposed to a standard culture medium, DMEM (Figure S4). This control showed that fibroblasts lost the activation pattern and came back to the quiescent state before activation. Together, these results showed not only a different gene expression pattern on fibroblasts when they were exposed to the “malignant signaling” of CM-EMC and the “benign signal” from the normal bladder cultures but also that activated fibroblasts by CM-EMC could be re-programmed into a “deactivated” fibroblasts by adding the “benign signaling” from normal bladder cultures or to a quiescent fibroblasts by adding a standard culture medium without the enrichment of growth factors present in the conditioned medium.

## 4. Discussion

We present a three-dimensional in vitro model to recreate bladder carcinoma in vitro. It has previously been shown that normal healthy epithelial cells lose their differentiated phenotype when grown in standard 2D cultures, though not if they are incorporated into a complex stroma-containing organoid [9].

Vayusin I. et al. summarized models of bladder organoids and spheroids using different methods and cell sources. According to this review, our model falls into the group of “bladder carcinoma plus bladder stroma” [24]. There, they only reported one study made with primary carcinoma cells that would be relevant for comparison with our model [24,25]. However, we must highlight that the scaffold that we presented here aimed not only to preserve the protein composition but also to conserve the structural protein microarchitecture found in the natural bladder. Similar scaffolds using native bladder tissue have been developed, but the protocols of decellularization are different and for long periods of time or the matrices are lyophilized and reconstituted [24]. Here, we did not lyophilize our scaffolds, and the decellularization process was gentle and short in order to avoid damage to the protein composition and, most importantly, structure. All these features of our scaffold made our model unique because it kept both the three-dimensional structure and the native protein organization of the extracellular matrix, thus giving support to the cells seeded on it and allowing them to organize themselves into their specific places.

Mullenders J. et al. reported the characterization of the different phenotypes of primary urothelial cells from bladder carcinomas from mice and humans grown as organoids [26]. The main difference between our models is that Mullenders J. et al. used a basement membrane extract as their extracellular matrix and grew and maintained the organoids with the addition of external growth factors. The basement membrane is an essential structure part the extracellular matrix in direct contact with epithelial cells. Even the authors’ model was amazing, another fundamental difference was that they obtained a differentiated epithelium using primary cells and managed to culture them for long periods by using external growth factors included in the culture medium (e.g., FGF2, FGF7, and FGF10) [26]. Our decellularized scaffolds not only contained the basement membrane’s proteins and structure but also conserved the other protein components and the structure of the whole native extracellular matrix, as mentioned above. We consider that the preservation of this complex protein network of the extracellular matrix is key to allow for the maintenance of the cells seeded on these scaffolds for long periods and without the addition of external growth factors. We believe that the advantage of our model is based on our unique scaffold that aims to preserve the protein and structural composition of the native organ-specific matrix. This feature not only gives structural support that allows cell organization but also keeps the organ specific protein signaling between the noncellular components of the native ECM and the cells seeded on it, which results in the maintenance of the cell proper functions. Of course, we are aware that stromal cells were not included in the present work, but our scaffolds enable one to include stromal immune and nonimmune cells in future studies.

Using tissue-specific scaffolds, previous works in our laboratory have shown that normal primary cells not only grow and form the typical epithelium of the original organ but also express and secrete tissue-specific proteins [16,17,18]. Surprisingly, we found that contrary to normal cells, carcinoma cells displayed a similar gene expression pattern of the six gene tested, when the cells were seeded on the scaffolds or when the cells were grown “naked” on standard monolayer plastic cultures.

Our model allowed us to further analyze the factors secreted by the carcinoma cells in an in vivo-like environment. The analysis of the conditioned medium showed a different pattern of the secreted growth factors VEGF, FGF, and PIGF, which were secreted at significantly higher levels in the CM of EMC cultures compared with the CM of same carcinoma cells cultured on plastic. VEGF, FGF, and PIGF have been related to excessive cell proliferation, angiogenesis, and the modulation of inflammatory response implicated in tumor development and progression [22,27,28]. Specifically, PIGF has been reported to be a mediator of the immune response led by VEGF during carcinogenesis [12,28]. Additionally, it has been pointed out that PIGF is directly involved in triggering IL-6 production via a calcineurin-dependent pathway and the proliferation of fibroblasts [22,29]. In bladder carcinoma, these three growth factors have been found to be in higher concentrations in urine and plasma from patients than those of healthy donors [30,31]. Our results suggest that primary carcinoma cells preserve the “secreted signature” of the carcinoma cells in vivo when they are seeded on bladder scaffolds but not when they are seeded on plastic. It must be pointed out that we only addressed six growth factors, but it is clear that a conditioned derived medium contains a broad repertoire of secreted proteins in specific concentrations and combinations [32] that can orchestrate tumor progression or a tumor-suppressive response. It has been reported that the wide repertoire and multifunctionality of growth factors and cytokines present in the tumor microenvironment modulates the phenotype of stromal cells such as fibroblasts [12,13,14].

Here, we found that the conditioned medium from the same EMC cultures could induce in adjacent stromal fibroblasts a pro-inflammatory phenotype or a myofibroblast phenotype depending on the concentration. Previously, some studies had induced the activation of different phenotypes of fibroblasts using a conditioned medium from 3D cultures of cancer cells [13,14,33]. Those studies also showed that depending on the method of the culture, i.e., Matrigel or monolayer, activated fibroblasts show a different phenotype. Unlike these studies, our system allowed us to switch between two different activated fibroblast phenotypes, even when they were seeded in monolayer under the same culture conditions but with changing concentrations of the conditioned medium, so these results support the plasticity of activated fibroblasts.

Between the GFs involved in the activation of fibroblasts, it has been reported that FGF and PDGF can modulate fibroblast function [12,34,35]. We hypothesized that a specific combination of GFs and not only one molecule or growth factor is involved in this modulation. Here, we found not only FGF, PDGF but also VEGF and PIGF in the CM of EMCs. We suggest that the PIGF secreted by carcinoma cells could also be part of the activation signaling of fibroblasts.

Following our results that displayed that a paracrine signaling of carcinoma cells in a proper context could activate fibroblasts, we hypothesized that paracrine signaling from a normal tissue would not induce this activation. We found that the gene expression of fibroblasts exposed to CM from cultures of normal tissue was significantly different from the two activated fibroblast phenotypes induced by EMC cultures. Additionally, we hypothesized that if fibroblasts could interconvert between activated phenotypes, they could also be reprogrammed to return to a normal phenotype. Previously, other authors suggested that activated fibroblasts can switch between phenotypes depending on the stimulus received from the tumor microenvironment [12,36]. Additionally, it has been suggested that those different phenotypes of cancer-associated fibroblasts are different states of the activation of normal fibroblasts [36]. Here, we treated pro-inflammatory activated fibroblasts with CM from normal tissue. The results showed that the phenotype of pro-inflammatory-activated fibroblasts was the reverse of the same phenotype of fibroblasts treated only with CM from normal tissue.

Many studies have demonstrated the heterogeneity of activated fibroblasts in the tumor microenvironment [12,13,36]. Recent works have shown how fibroblasts can interconvert between the pro-inflammatory and myofibroblast phenotypes [14]. However, the heterogeneity, stability, and reversibility of those states need to be studied in depth, and CAFs have recently been postulated as a potential target for cancer therapy. Specifically, some signaling pathways—i.e., FGFR, TGFβ, and Hedgehog, which have been shown to be related to the modulation of CAF phenotypes—are currently being investigated [12].

## 5. Conclusions

Altogether, the results showed that for modelling carcinoma in vitro, it is important to preserve not only the interaction between the epithelium and extracellular matrix but also the microarchitecture and protein complexity of the extracellular matrix. Our 3D bladder carcinoma model allowed us to study the secreted signaling of primary carcinoma cells and its effect on stromal fibroblasts. Here, we displayed not only that the fibroblasts can switch between activated phenotypes depending on the concentration and combinations of the growth factors secreted by cancer epithelial cells using our 3D system but also that a benign signaling from normal epithelial cells could “deactivate” the pro-inflammatory phenotype in this case. This work provides a platform that allows for the study of the paracrine signaling pathways of malignant and benign tissues, and it proposes a model where different therapeutic modalities can be tested.

## Data Availability

The data presented in this study are available in this article (and supplementary material).

## Supplementary Materials

The following are available online at https://www.mdpi.com/article/10.3390/cancers13122972/s1. Figures S1: Continuation of Characterization of the extracellular matrix from bladder decellularized matrices. Figure S2: Characterization of human primary urothelial cells from bladder carcinoma seeded on the scaffolds. Figure S3: Normal fibroblasts exposed to 100% of conditioned medium from different days of bladder-EMC cultures and cancer epithelial cells seeded on plastic. Figure S4: Fibroblast activated with 100% of EMC-CM came back to “quiescent” state after exposed to standard culture medium DMEM 10% FCS. Table S1: Classification of bladder carcinoma of the samples used.
