# Supplementary material for: Paracrine Signaling from a Three-Dimensional Model of Bladder Carcinoma and from Normal Bladder Switch the Phenotype of Stromal Fibroblasts
Source: Cancers (Basel). 2021 Jun 14;13(12):2972. doi: 10.3390/cancers13122972 (PMC8231763; doi:10.3390/cancers13122972)
Supplement: Supplementary file 1 [file cancers-13-02972-s001.zip › cancers-1230150-supplementary.pdf]

Supplemental Material Paracrine Signaling from a Three-Dimensional Model of Bladder Carcinoma and from Normal Bladder Switch the Phenotype of Stromal Fibroblasts

Sandra Camargo, Ofer N. Gofrit, Assaf Assis and Eduardo Mitrani

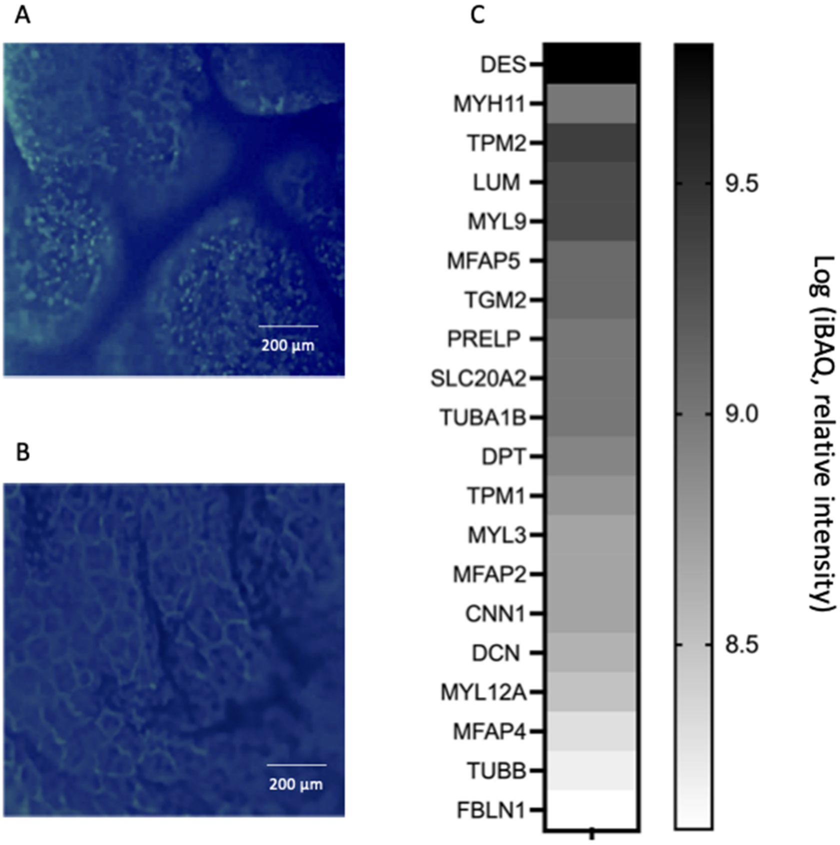

Figure S1. Continuation of Characterization of the extracellular matrix from bladder decellularized matrices. DAPI staining of pig bladder before (A) and after (B) decellularization. (C) The most abundant proteins from the extracellular matrix of the bladder scaffolds with iBAQ ≥ 8, intensity-based absolute quantification.

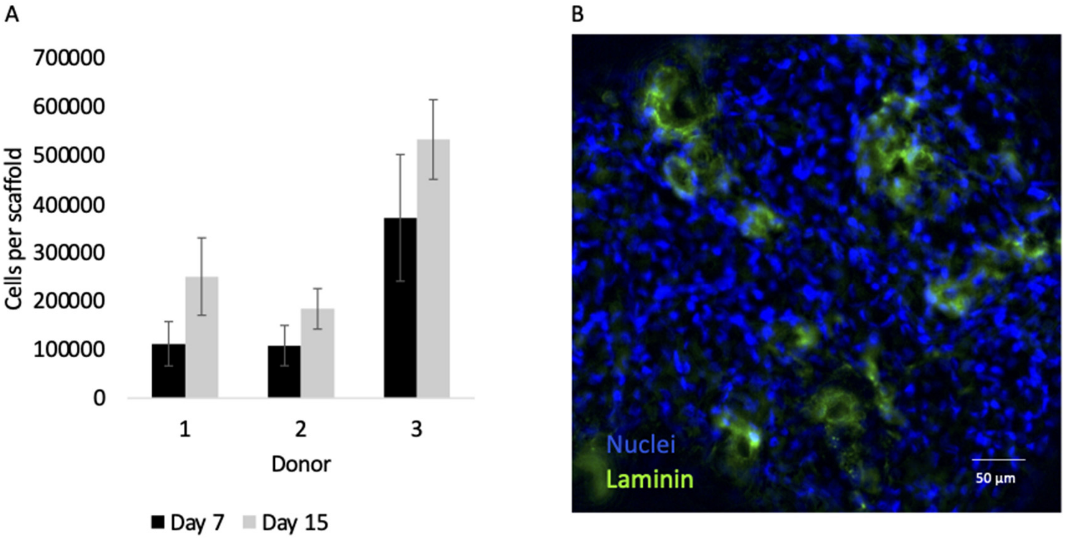

Figure S2. Characterization of human primary urothelial cells from bladder carcinoma seeded on the scaffolds. (A) Cell concentration per scaffold. Donor 1 donor 2 and donor 3, at days 7 and 15. (B) Top view of human primary urothelial cancer cells (nuclei in blue stained with DAPI) grown on the basement membrane of the scaffold (Immunofluorescence staining in green for Laminin present in the basement membrane of the scaffold), Day 15. Scale bar 100 µm.

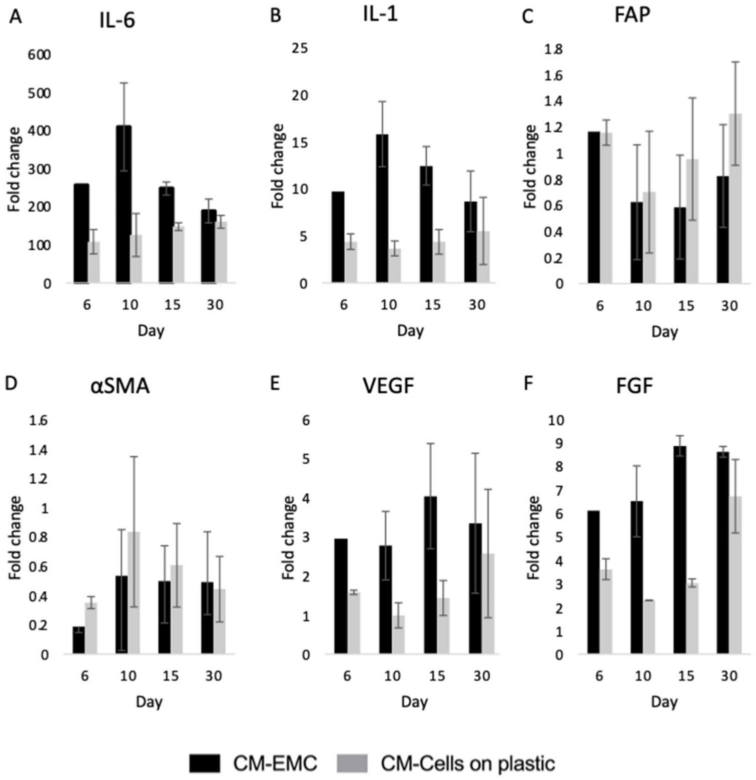

Figure S3. Normal fibroblasts exposed to 100% of conditioned medium from different days of bladder-EMC cultures and cancer epithelial cells seeded on plastic. (A) IL-6 and (B) IL-1. A myofibroblast phenotype with up regulation of (C) FAP, (D) αSMA, (E) VEGF and (F) FGF.

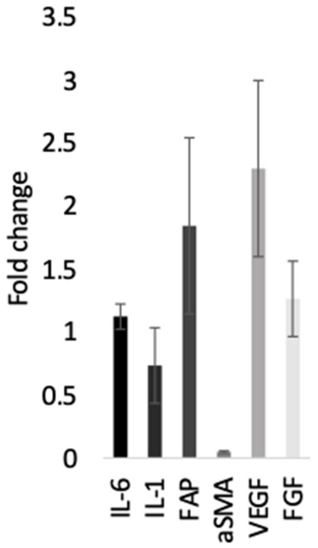

Figure S4. Fibroblast activated with 100% of EMC-CM came back to "quiescent" state after exposed to standard culture medium DMEM 10% FCS. Gene expression of IL-6, IL-1, FAP, αSMA, VEGF and FGF. The fold change was calculated in comparison with fibroblasts cultured in DMEM 10% FCS.

Table S1. Classification of bladder carcinoma of the samples used.

Donor	Type of bladder carcinoma
**1**	Ta high-grade
**2**	T1 high-grade
**3**	Ta low-grade
